# All-optical analog differential operation and information processing empowered by meta-devices

**DOI:** 10.1515/nanoph-2024-0540

**Published:** 2025-01-27

**Authors:** Chen Zhou, Yongtian Wang, Lingling Huang

**Affiliations:** Beijing Engineering Research Center of Mixed Reality and Advanced Display, MIIT Key Laboratory of Photonics Information Technology, School of Optics and Photonics, 47833 Beijing Institute of Technology, Beijing, 100081, China; National Key Laboratory on Near-surface Detection, Beijing, 100072, China

**Keywords:** optical analog computing, metasurface, edge detection, edge enhancement

## Abstract

The burgeoning demand for high-performance computing, robust data processing, and rapid growth of big data necessitates the emergence of novel optical devices to efficiently execute demanding computational processes. The field of meta-devices, such as metamaterial or metasurface, has experienced unprecedented growth over the past two decades. By manipulating the amplitude, phase, polarization, and dispersion of light wavefronts in spatial, spectral, and temporal domains, viable solutions for the implementation of all-optical analog computation and information processing have been provided. In this review, we summarize the latest developments and emerging trends of computational meta-devices as innovative platforms for spatial optical analog differentiators and information processing. Based on the general concepts of spatial Fourier transform and Green’s function, we analyze the physical mechanisms of meta-devices in the application of amplitude differentiation, phase differentiation, and temporal differentiation and summarize their applications in image edge detection, image edge enhancement, and beam shaping. Finally, we explore the current challenges and potential solutions in optical analog differentiators and provide perspectives on future research directions and possible developments.

## 1 Introduction

The purpose of image processing is to improve or enhance the acquisition of information by the human eyes [1]. For input to any image processing system, analog or digital, is the light field. The output is captured by the detector, such as a camera, and may be digitally modified to capture the information of the light field. In the case where real-time manipulation of the optical field via optical elements is termed optical analog image processing, while the computational analysis of field intensity captured by a digital camera is known as digital or electronic image processing [2]. Despite the advent of deep learning-driven artificial intelligence ushering an information-intelligence era, it still faces various challenges for the storage and real-time processing of massive data [3], [4], [5], [6], [7]. Compared with traditional digital or electronic computing, spatial optical analog computing has gained increasing attention in realizing mathematical operations and information processing, because of its inherent parallel processing capability, high computational speed, and low loss [8], [9], [10], [11], [12], [13], [14], [15], [16], [17], [18], [19]. In particular, optical differential operations allow for edge detection or edge enhancement of target image objects, leading to fast recognition and compression of the object’s feature information [20], [21], [22], [23]. It is crucial for the development of a variety of modern technologies including augmented and virtual reality (AR/VR), biomedical imaging, and autonomous driving [24], [25], [26].

Generally, there are two kinds of methods for spatial optical analog differentiation, namely spatial Fourier transfer approach and Green’s function (GF) approach [27]. The theoretical foundation of spatial Fourier transfer approach is often referred to as the 4*f* filtering system. It can be dates back to the late 19th century when Abbe introduced his spatial imaging theory [28]. This concept is integral to understanding how optical systems process and manipulate light, particularly in the context of imaging and filtering. The spatial Fourier transfer approach, which consists of two lenses with a spatial filter in between, is used to perform Fourier transformations on light, allowing for differentiation operations and information filtering effects that can enhance image quality or extract specific information from complex optical fields. However, by reason of the heavy optical glass components, the whole system is relatively complex, and it is difficult to meet the current trends of minimization, hindering integration with compact nanophotonic circuits.

Recently, with the extraordinary development of advanced nanofabrication technologies, such as electron beam lithography (EBL) and focused ion beam (FIB), the thickness of various optical elements can be scaled down to the nanometer level. This enhances the interaction between light and matter, which is necessary for fully controlling the characteristics of incident light in miniaturized configurations. It also provides the possibility to address the long-standing issues of the 4*f* filtering system. A quintessential example is the planarized artificial meta-devices, including metamaterials and metasurface [29], [30], [31], [32]. Thanks to the optimized size, shape, orientation, and composition of subwavelength scale scatterers, metasurface is possible to efficiently controlling and flexibly manipulating the optical field [33], [34], [35], [36], [37], [38], [39], [40]. It is not only can be applied in the fields of multifunctional holography, spectral imaging, optical sensing, and quantum entanglement but also provide a powerful platform for realizing optical differentiation operations and image processing [41], [42], [43], [44], [45], [46]. In the early stages, the application of metasurface to spatial analog optical differentiation was mainly based on the 4*f* filtering concept, merely replacing the glass lens or filtering mask without fully demonstrating the powerful light field control capabilities of metasurface [27], [47], [48]. In 2014, Silva et al. [27] first proposed the concept of computational metamaterials and the Green’s function approach. They also explored the potential use of metamaterials for performing mathematical operations on images. The so-called Green’s function method refers to the direct design and optimization of optical devices in the spatial domain to meet specific spatial frequency domain transfer functions. This method can directly implement various analog optical computing functions, including analog optical differentiation, in the spatial domain. Afterward, utilization of metasurface to realize differential operations and various image edge detection or edge enhancement has been continuously proposed, greatly enriching the application of meta-devices in image processing.

**Figure 1:**
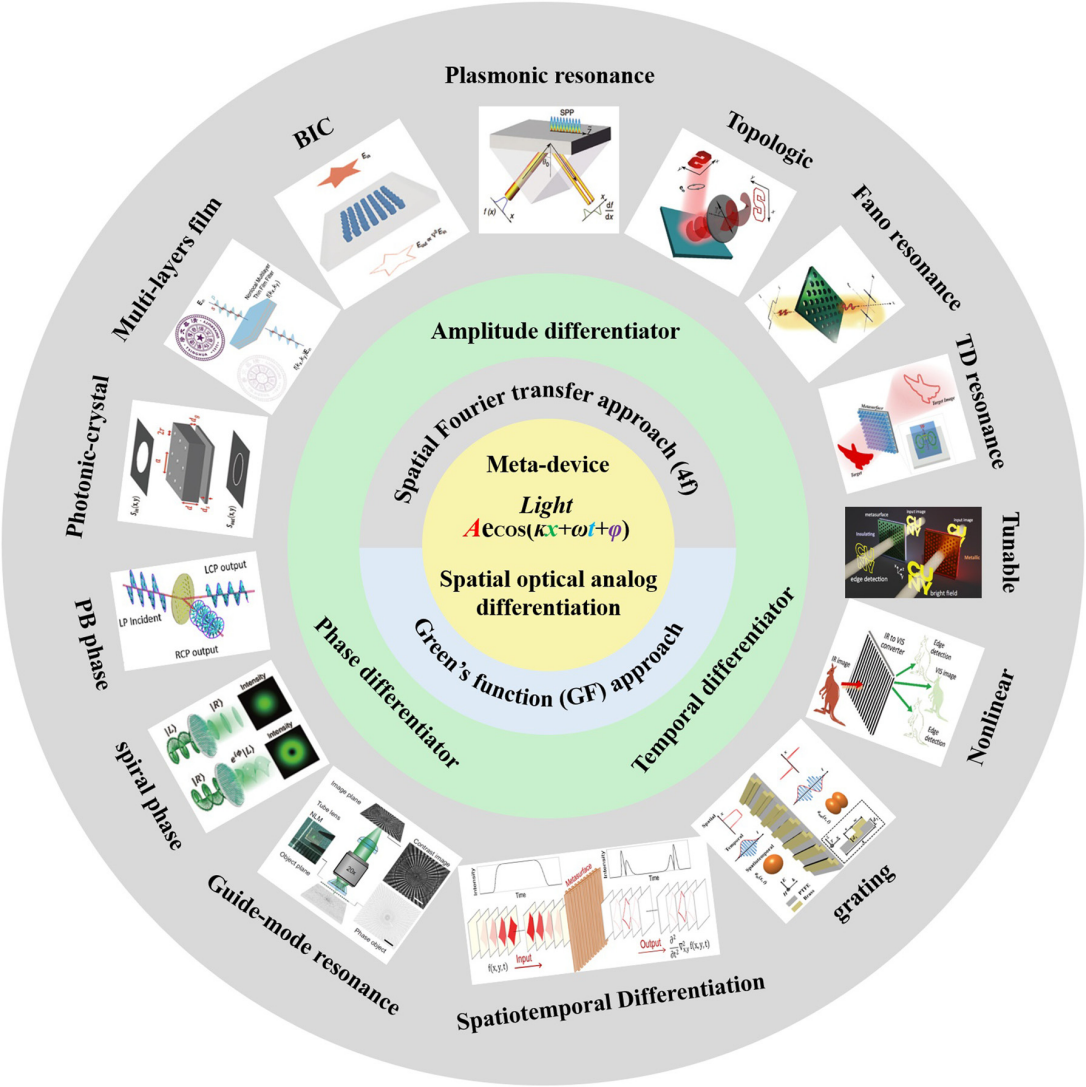
Spatial optical analog differentiator: from principle to applications and advances. Reproduced from Refs. [15], [20], .

Here, we provide a comprehensive review of the mechanism, research progress, and applications of spatial optical analog differentiator (Figure 1). Firstly, we introduce the mathematical and physical principles of meta-device differentiators based on two realization methods. Then, we discuss recent advances in information processing of optical fields in terms of amplitude, phase, and temporal by spatial analog optical differentiators based on metasurface. In addition, the latest advancements in dynamic modulation and nonlinear modulation of spatial analog optical differential devices are also reviewed. Finally, we present some challenges facing spatial analog optical computing devices and analyze an outlook from several directions, including innovative design strategies, manufacturing processes, material performance, and dynamic control.

## 2 Fundamental principles

### 2.1 Fourier domain filtering approach

**Figure 2:**
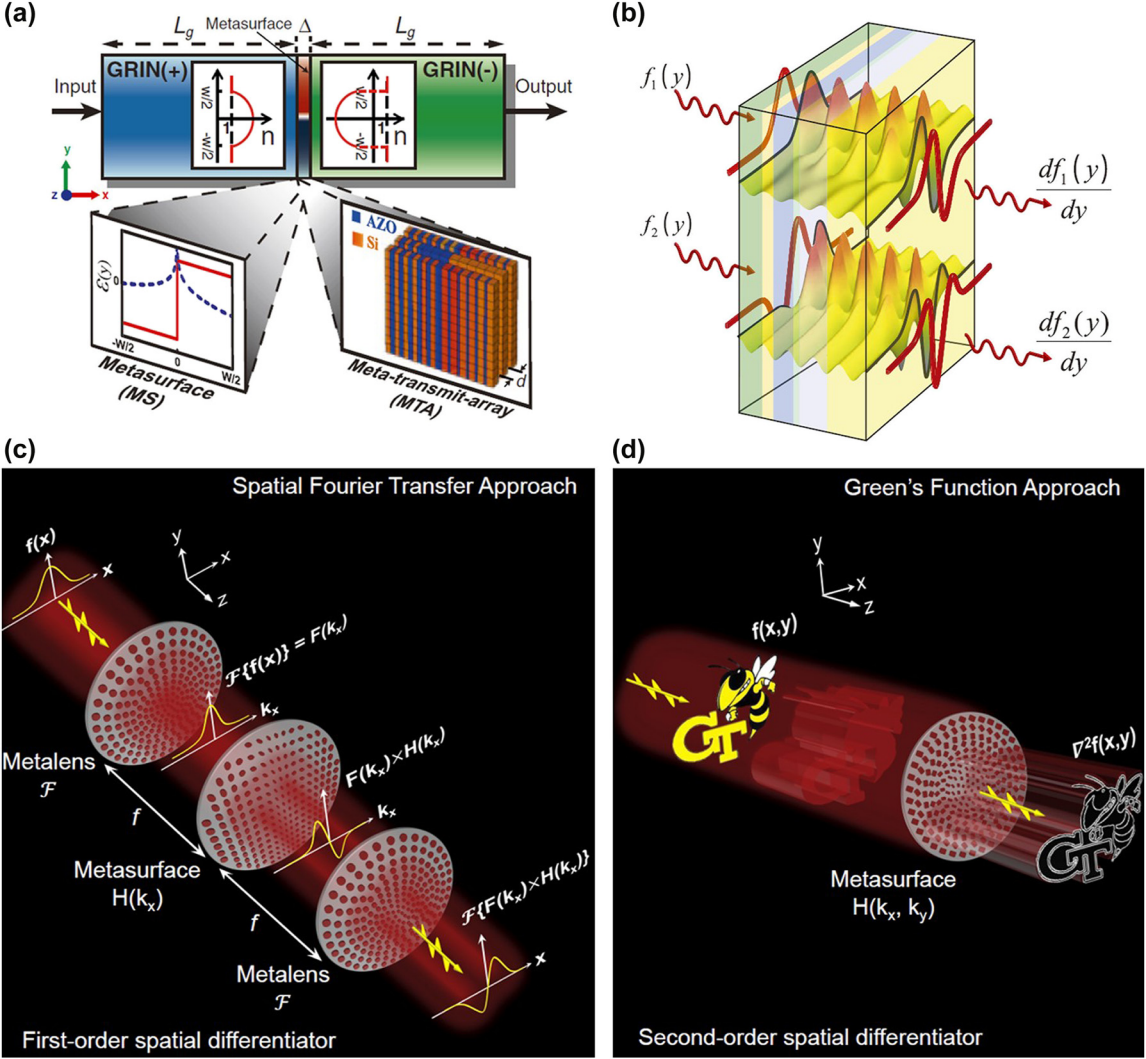
Metamaterials for spatial analog computing. (a) Designed cascaded GRIN(+)/metastructure/GRIN(−) system to perform mathematical operations in the spatial Fourier domain. Reproduced from Ref. [27]. (b) A conceptual sketch of computational metamaterials. Reproduced from Ref. [27]. (c) Implement first-order spatial differentiation using a metasurface based on the spatial Fourier transfer approach. Reproduced from Ref. [61]. (d) Implement second-order two-dimensional Laplacian differentiation using a metasurface based on the Green’s function (GF) approach. Reproduced from Ref. [61].

Figure 2a illustrates a schematic diagram of a spatial analog optical computing device designed using cascaded metamaterials proposed by Silva et al., which is essentially based on the concept of a 4*f* filtering system. The innovation lies in the fact that the planar graded index (GRIN) lens is used to replace the traditional refractor lens. The spatial frequency filter is implemented by optically transparent materials such as metasurface or metasurface-array. For a 4*f* system with linear transverse invariance, the relationship between the incident light field $E_{\text{in}}\left(x,y\right)$ and the outgoing light field $E_{\text{out}}\left(x,y\right)$ is represented by the following convolution relationship [27]:

(1) $$E_{\text{out}}\left(x,y\right)=h\left(x,y\right)*E_{\text{in}}\left(x,y\right)$$

where $h\left(x,y\right)$ is the impulse response function of the system.

Using Fourier transform, in the spatial frequency domain, Equation (1) can be expressed as:

(2) $${\tilde{E}}_{\text{out}}\left(k_{x},k_{y}\right)=H\left(k_{x},k_{y}\right){\tilde{E}}_{\text{in}}\left(k_{x},k_{y}\right)$$

where, ${\tilde{E}}_{\text{out}}\left(k_{x},k_{y}\right)$ , $H\left(k_{x},k_{y}\right)$ , and ${\tilde{E}}_{\text{in}}\left(k_{x},k_{y}\right)$ correspond to the spatial Fourier transform of $E_{\text{out}}\left(x,y\right)$ , $h\left(x,y\right)$ , and $E_{\text{in}}\left(x,y\right)$ , respectively. It can be seen that by engineering the resonance, polarization, phase, and orbital angular momentum characteristics of metasurface, one can construct distributions that meet the requirements of $H\left(k_{x},k_{y}\right)$ or $h\left(x,y\right)$ in either Fourier space or real space. This enables the realization of optical analog differential computing functions, which can be applied to edge detection in images. Traditionally, this is achieved using masks with specific light transmission patterns, but now it can be implemented using advanced and compact metasurface.

### 2.2 Green’s function approach

In order to directly perform mathematical operations in the spatial domain, Silva et al. conceived a type of computational metamaterial, as shown in Figure 2b. When any wave signal propagates through it, such metamaterials can carry out the required mathematical operations, such as spatial optical simulation of differential computation. This method is referred to as the Green’s function method. Compared to the 4*f* system method, the Green’s function method does not require Fourier transform lenses to convert between the spatial domain and spatial frequency domain for the incident and outgoing light fields. It only requires that the metamaterials or metasurface satisfy the optical transfer function (OTF) necessary for differential operations or other mathematical computations. This type of OTF can be directly implemented in the spatial domain by constructing meta-devices with reflection and transmission responses related to the angle of incidence. Common structures of OTFs required for constructing optical analog differentiators include gratings, multilayer films, photonic crystal slabs, and various metasurface, which will be introduced in detail in this review.

### 2.3 The order of spatial differentiator

For a two-dimensional nth-order spatial analog differentiator, the relationship between the output and the input optical field distribution in spatial is expressed as follows:

(3) $$\begin{array}{ll} E_{\text{out}}\left(x,y\right) & =\frac{\partial^{n}}{\partial x^{n}}E_{\text{in}}\left(x,y\right)+\frac{\partial^{n}}{\partial y^{n}}E_{\text{in}}\left(x,y\right)=\nabla^{n}E_{\text{in}}\left(x,y\right) \end{array}$$

where, *n* is an integer. Equation (3) can be derived based on the Fourier transform formula as follows:

(4) $${\tilde{E}}_{\text{out}}\left(k_{x},k_{y}\right)=\left[{\left(ik_{x}\right)}^{n}+{\left(ik_{y}\right)}^{n}\right]{\tilde{E}}_{\text{in}}\left(k_{x},k_{y}\right)$$

then, the expression for the optical transfer function of a two-dimensional nth-order spatial optical analog differential operator is:

(5) $$H\left(k_{x},k_{y}\right)=\frac{{\tilde{E}}_{\text{out}}\left(k_{x},k_{y}\right)}{{\tilde{E}}_{\text{in}}\left(k_{x},k_{y}\right)}={\left(ik_{x}\right)}^{n}+{\left(ik_{y}\right)}^{n}$$

If differentiation is only required along a single direction, either *x* or *y*, the transfer function only needs to take the form of ${\left(ik_{x}\right)}^{n}$ or ${\left(ik_{y}\right)}^{n}$ . When *n* = 1, it corresponds to an odd-order differentiator, indicating that the amplitude of the optical differentiator’s transfer function exhibits a linear relationship with spatial frequency, that is, $H\left(k_{x},k_{y}\right)\propto ik_{x}+ik_{y}$ , and there is a variation of *π* in phase, as shown in Figure 2c [61]. When *n* = 2, it corresponds to an even-order differentiator, indicating that the amplitude of the optical differentiator’s transfer function exhibits a quadratic relationship with spatial frequency, that is, $H\left(k_{x},k_{y}\right)\propto -\left(k_{x}^{2}+k_{y}^{2}\right)$ , while the phase remains constant. At this point, the metasurface acts as a Laplacian differential operator ∇^2^ = *∂* ^2^/*∂x* ^2^ + *∂* ^2^/*∂y* ^2^, capable of obtaining edge information in all directions of the image, which holds significant value for applications in the field of image processing, as shown in Figure 2d.

### 2.4 The principles of temporal differentiator

For an optical pulse signal with a carrier angular frequency of *ω*_0_, the complex amplitude envelope of the signal is $e_{\text{in}}\left(t\right)$ . The input optical field can be expressed as [62]:

(6) $$E_{\text{in}}\left(t\right)=e_{\text{in}}\left(t\right)\exp\left(-i\omega_{0}t\right)$$

the Fourier transform of the time-domain optical field $E_{\text{in}}\left(t\right)$ is given by:

(7) $$E_{\text{in}}\left(\omega \right)=\int e_{\text{in}}\left(t\right)\exp\left[i\left(\omega -\omega_{0}\right)t\right]\text{d}t$$

where *ω* is the angular frequency of the optical field signal. Similar to spatial linear time-invariant systems, considering the linear time-invariant characteristics, the output optical signal $E_{\text{out}}\left(t\right)$ of the optical system can be obtained through the convolution of the input optical pulse signal $E_{\text{in}}\left(t\right)$ and the system’s time impulse function $h\left(t\right)$ .

(8) $$E_{\text{out}}\left(t\right)=h\left(t\right)*E_{\text{in}}\left(t\right)$$

The spectral transformation relationship between the output signal and the input signal can be described by the frequency-domain transfer function $H\left(\omega \right)$ . $H\left(\omega \right)$ is the Fourier transform of the time impulse function.

When the complex amplitude envelope $e_{\text{out}}\left(t\right)$ of the output pulse is the integer n-th order derivative of the input optical signal’s complex amplitude $e_{\text{in}}\left(t\right)$ , the optical field of the output signal can be expressed as:

(9) $$E_{\text{out}}\left(t\right)=\frac{\partial^{n}e_{\text{in}}\left(t\right)}{\partial t^{n}}\exp\left(-i\omega_{0}t\right)$$

At this point, according to the time Fourier transform, it can be deduced that the angular frequency distribution of the output optical field is:

(10) $$E_{\text{out}}\left(\omega \right)=\frac{\partial^{n}e_{\text{in}}\left(t\right)}{\partial t^{n}}\exp\left[i\left(\omega -\omega_{0}\right)t\right]\text{d}t$$

Therefore, for an ideal optical time n-th order integer differentiation operation, the transfer function in the frequency domain is:

(11) $$H\left(\omega \right)=\frac{E_{\text{out}}\left(\omega \right)}{E_{\text{in}}\left(\omega \right)}={\left[i\left(\omega -\omega_{0}\right)\right]}^{n}$$

Consequently, it can also be deduced that the amplitude response of the optical field time differentiator is zero at the signal’s carrier frequency, and there is a phase jump of *nπ* in the phase. In optical time signal processing, since it is necessary to control the angular frequency information of the signal, resonant devices are typically used to construct the required linear spectral response to achieve time differentiation processing.

## 3 Amplitude differentiator and edge detection

### 3.1 Amplitude differentiator based on spatial Fourier transfer approach

**Figure 3:**
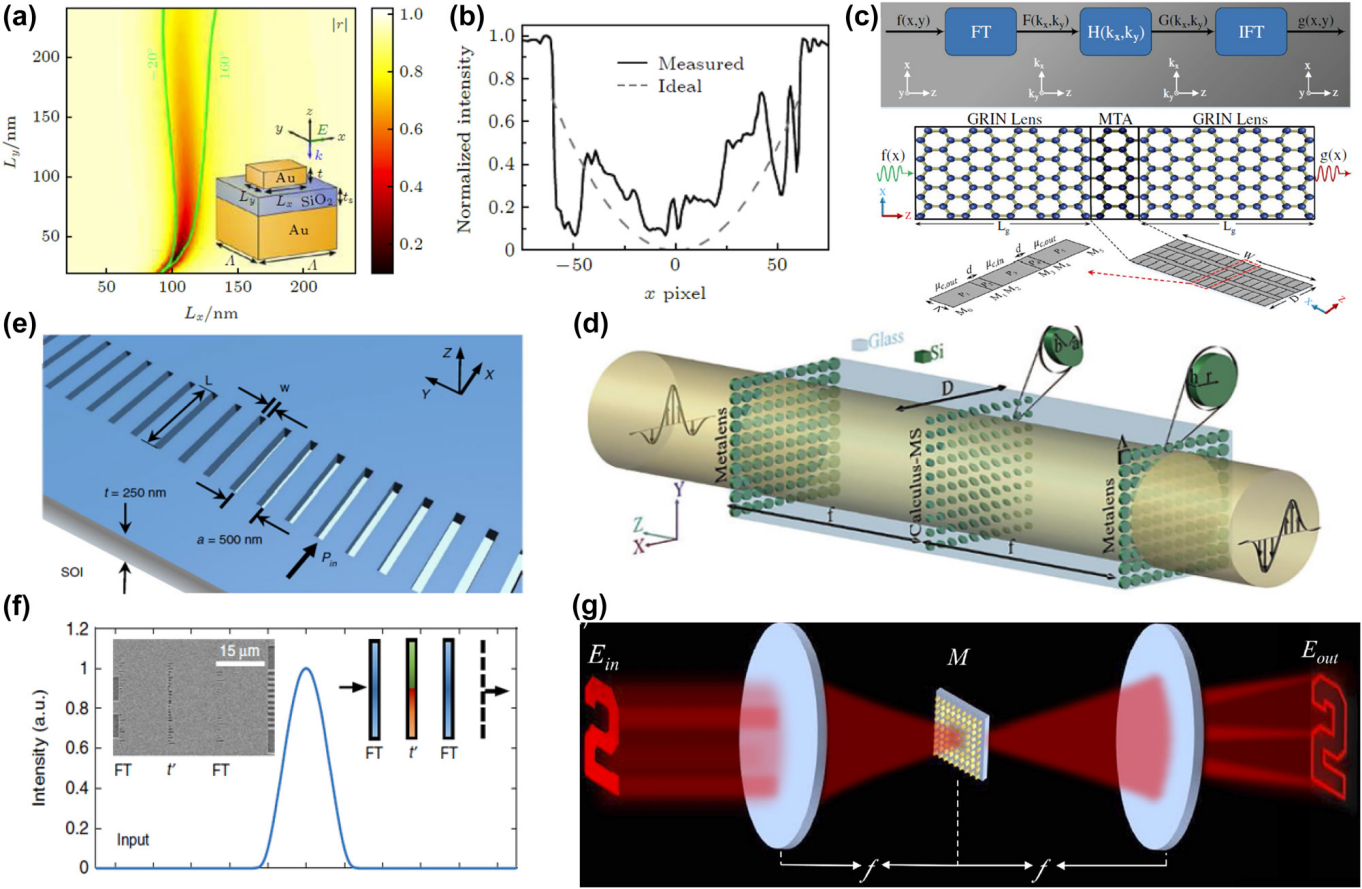
Amplitude differentiator based on conventional spatial Fourier transfer approach. (a) and (b) Reflective plasmonic metasurface for differentiation operation. Reproduced from Ref. [47]. (c) Graphene-based metasurface for differentiation operation. Reproduced from Ref. [63]. (d) Metalens/calculus-MS/metalens blocks for differentiation operation. Reproduced from Ref. [48]. (e) and (f) On-chip analog differentiation operation based on a Si-on-insulator (SOI) platform. Reproduced from Ref. [64]. (g) A schematic of differentiation operation and edge detection based on the polarization-correlated interference induced by tetratomic metasurface with dual geometric phases. Reproduced from Ref. [65].

With the advancement of micro-nanofabrication technology, using metasurface to replace components in 4*f* systems for spatial optical analog differentiation and edge detection, as well as to enhance the integrability of the entire system, has been successively proposed. Figure 3a illustrates the gap-surface plasmon resonance mode utilized by Pors et al. [47] within a metal–insulator–metal (MIM) structure. By altering the lateral dimensions of the gold nanorod resonators on the top layer, independent control over the reflection phase and amplitude at 800 nm near-infrared light is achieved, including 0 and *π* phase shifts. Although there are discrepancies between the experimental results and theoretical predictions due to fabrication errors, as shown in Figure 3b. However, these metamaterial-based structures not only enable spatial domain analog optical differentiators but also can be utilized as optical analog integrators. This dual functionality allows for advanced optical computing applications.

In response to the urgent demand for highly miniaturized computing systems in real-life applications, Abdollahramezani et al. [63] demonstrated the dynamic control over the amplitude and phase distribution of propagating plasmons in graphene, as shown in Figure 3c. They achieved good optical micro-differentiation effects in simulations. The ultra-thin nature of graphene provides a potential platform for the design of chip-scale optical micro-differentiators. Due to the inherent ohmic losses and low coupling efficiency associated with metallic materials, Sajjad et al. [48] designed a metasurface composed of an array of silicon disks (Figure 3d). By leveraging the interplay between electric/magnetic resonances and the concept of 4*f* filtering, they implemented devices capable of performing differentiation operations and solving ordinary differential equations with constant coefficients.

Considering the mature wafer-based silicon photonics, the implementation of on-chip mathematical operators is expected to enable the next generation of compact, low-power, and versatile computational photonic integrated circuits. Wang et al. [64] demonstrated that an on-chip one-dimensional high-contrast transmit-array of metasurface, as shown in Figure 3e, can achieve on-chip differentiation operations (as depicted in Figure 3f) and other signal processing functionalities. Utilizing the 4*f* filtering concept, by designing the width and length of the slits in the silicon substrate, complete control over the transmission amplitude and phase distribution can be achieved over a broad bandwidth. This integrated metalens significantly reduces the overall size of on-chip photonic processors. In addition, Bi et al. [65] designed a tetratomic macropixel metasurface based on dual geometric phase modulation and polarization-correlated interference, as shown in Figure 3g. It is capable of achieving target edge detection under visible wavelengths and even under white light conditions. This addresses the application limitations of optical edge detection based on traditional 4*f* filtering systems in terms of wide bandwidth, multiple wavelengths, and white light conditions. Recently, Bi et al. [66] has also utilized the Pancharatnam–Berry (PB) phase to design a polarization multiplexing metasurface capable of performing second-order two-dimensional differentiation and integration operations simultaneously. This enables edge enhancement and denoising on images at multiple visible wavelengths. Such concurrent processing architecture offers a promising pathway for multifunctional and higher-speed image processing applications.

Although the concept and theory of computational metasurface were proposed quite early. Unfortunately, the experimental implementation of optical edge detection using metamaterials and metasurface remains challenging. Therefore, researchers proposed the idea to combine the 4*f* filtering concept with the spin Hall effect of light (SHEL) to achieve optical differentiation and image edge detection [67]. The SHEL means that when light interacts with an optical material, due to the mutual coupling of spin angular momentum and orbital angular momentum, the left-handed circularly polarized (LHCP) and right-handed circularly polarized (RHCP) components of the outgoing light undergo opposite transverse displacements [68], [69], [70], [71], [72]. Considering the case where the displacement distance is sufficiently small, the overlapping central part contains both LHCP and RHCP components. The orthogonal polarization component in the output light, relative to the input linearly polarized light, will be the result of a first-order differential operation on the input light field. When the displacement is much smaller than the scale of image details, the first-order difference can be approximated as a first-order derivative.

**Figure 4:**
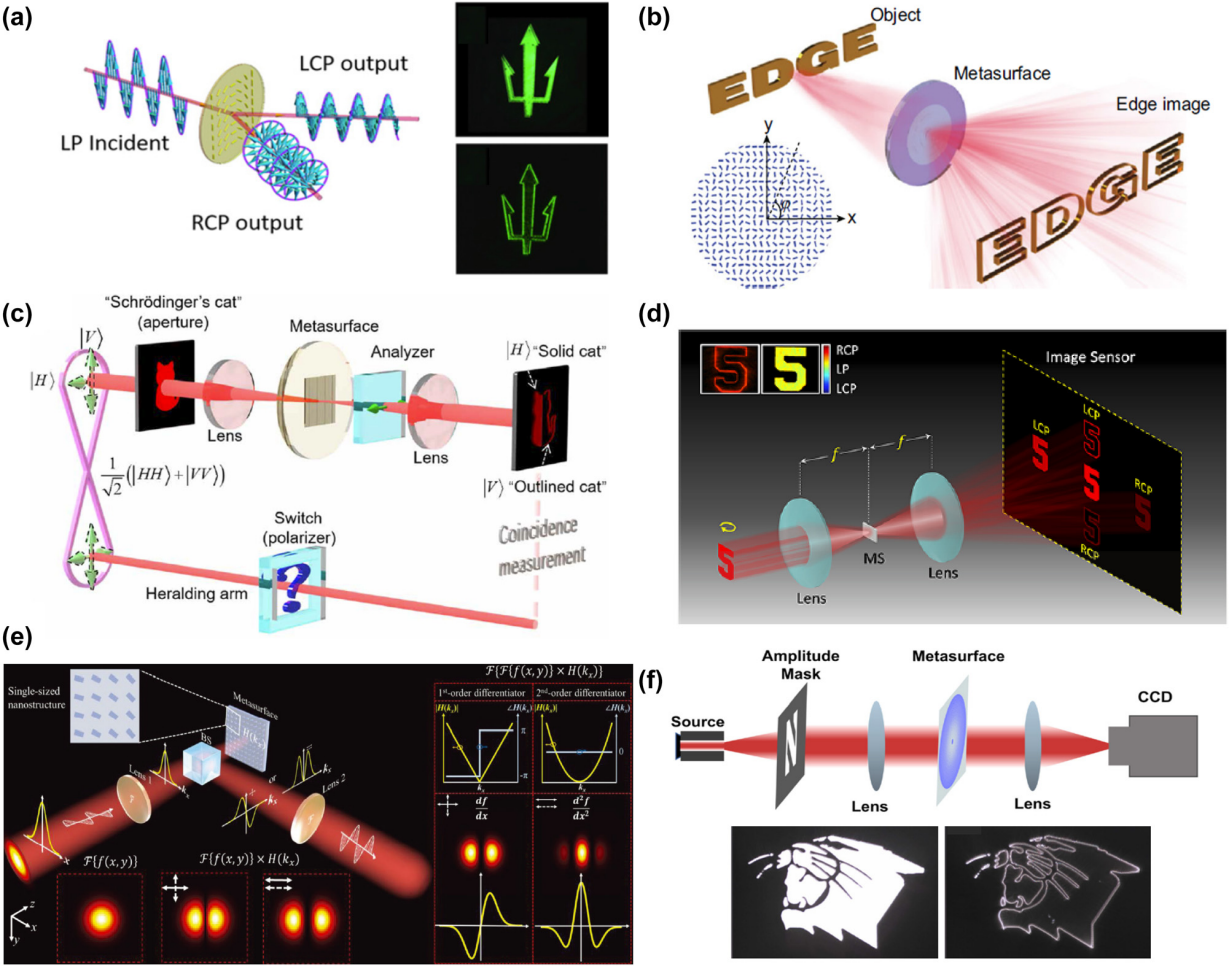
Amplitude differentiator based on creativity spatial Fourier transfer approach. (a) Differentiation operation based on dielectric PB-phase metasurface. Reproduced from Ref. [49]. (b) Two-dimensional spatial differentiation based on two-dimensional dielectric PB-phase metasurface. Reproduced from Ref. [73]. (c) Quantum edge detection based on dielectric PB-phase metasurface. Reproduced from Ref. [74]. (d) Simultaneously achieves spatial differentiation operations and polarization control based on a multifunctional metasurface. Reproduced from Ref. [75]. (e) Multiplexed differentiators based on Malus metasurface. Reproduced from Ref. [76]. (f) Broadband differentiation operation based on multiple illumination environments. Reproduced from Ref. [77].

In 2019, Zhou et al. [49] utilized a phase gradient metasurface constructed with Pancharatnam–Berry (PB) phases to generate the spin Hall effect of light (SHEL) and successfully implemented high-efficiency first-order differentiation operations, as shown in Figure 4a. The experimental results demonstrated its high-resolution edge detection capability. To extend the edge detection dimension to two dimensions, Zhou et al. [73] further generalized the PB phase gradient to two dimensions, achieving broadband two-dimensional spatial differentiation, as depicted in Figure 4b. Additionally, based on the work as shown in Figure 4a, Zhou et al. [74] innovatively utilized the switching mode of a polarization-entangled photon source, to introduce the concept of switching between bright-field imaging and edge detection, as shown in Figure 4c. By remotely toggling the entangled photon pairs and manipulating the polarization state of the trigger photon, they were able to achieve different imaging effects for bright-field images and one-dimensional edge images. This approach opens up a new platform for optical spatial differentiation in the field of quantum imaging.

Typically, edge enhancement and polarization detection hold significant value in the biomedical field. However, currently available microscopic imaging systems are limited to performing only single function. Intaravanne et al. [75] developed a multifunctional metasurface microscope based on spatial differentiation operations and polarization multiplexing, as depicted in Figure 4d. This microscope is capable of obtaining five images with different optical characteristics on the same imaging plane simultaneously, including edge detection and bright-field imaging, as well as polarization imaging. Generally speaking, once an optical differentiator based on a 4*f* filtering system is designed, it is typically limited to performing a single differentiation function. However, Liang et al. [76] have designed a Malus metasurface consisting of single-sized nanostructures, as illustrated in Figure 4e. By adjusting the in-plane orientation angle of the nanostructure and polarization direction, it is capable of achieving both first- and second-order differentiation operations, as well as the corresponding image edge detection. This advancement enhances the capabilities of optical differentiation devices without incurring additional complexity in design or increasing costs in nanofabrication processes.

Considering the complexity of the real application environment, it is urgent to have all-optical two-dimensional image processing equipment that can work under coherent and incoherent illumination conditions and can realize broadband, large NA, and multiple polarization channels. Tanriover et al. [77] designed a metasurface differentiator composed of aluminum ring resonators with varying widths on a SiO_2_ substrate, as shown in Figure 4f. It is capable of performing spatial differentiation and edge detection under various polarized coherent light and unpolarized incoherent light illumination conditions, while also achieving broadband differentiation effects. This is of significant value for practical applications such as autonomous driving.

### 3.2 Amplitude differentiator based on Green’s function (GF) approach

Building upon the Green’s function method proposed by Silva et al., it is feasible to directly utilize computational metamaterials to achieve spatial analog differentiation. Essentially, this requires an optical device that exhibits appropriate sensitivity to the angle of incidence, either in transmission or reflection, which provide direct filtering in the Fourier domain. This eliminates the need for additional optical elements and the associated propagation distances to access the Fourier plane. Such a phenomenon can be employed to perform direct edge detection and recognition of images in the spatial domain, rather than in Fourier space. In the natural world, obtaining angle-sensitive materials is often subject to numerous constraints. However, it is relatively straightforward to achieve angle-sensitive transmission or reflection spectra using meta-devices such as photonic crystals, metasurface, multilayer films, and diffraction gratings or meta-grating. This is attributed to their ability to create peculiar optical resonances or innovative optical effects. Subsequently, we primarily focus on resonance from the perspective of the Green’s function (GF) method, providing a comprehensive review of resonant systems for spatial amplitude differentiators.

**Figure 5:**
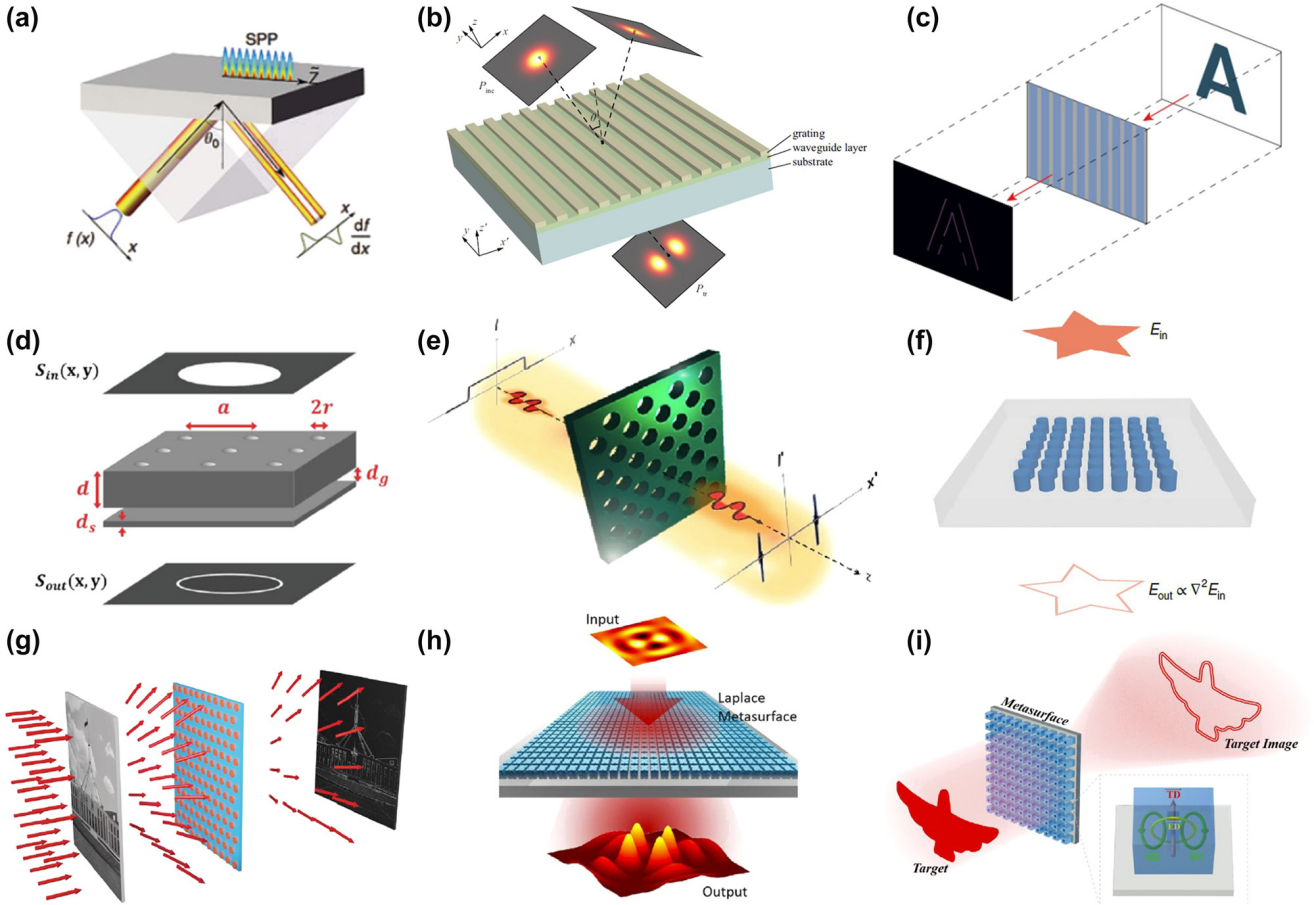
Resonant system of spatial amplitude differentiator based on Green’s function (GF) approach. (a) First-order spatial differentiation through excitation of SPP resonances on silver thin film. Reproduced from Ref. [50]. (b) Resonant diffraction grating performed the first-order spatial differentiation based on guided-mode resonance. Reproduced from Ref. [85]. (c) Dielectric metasurface performing first-order or second-order spatial differentiation based on Fano resonance. Reproduced from Ref. [86]. (d) Implementing a Laplacian differentiator using photonic crystal structures. Reproduced from Ref. [20]. (e) Schematics of nonlocal metasurface performing Laplacian differentiator. Reproduced from Ref. [51]. (f) Schematic of a photonic crystal slab acting as a Laplacian differentiator based on quasi-BIC. Reproduced from Ref. [52]. (g) Performing Laplacian differentiator based on Mie-resonances. Reproduced from Ref. [87]. (h) Performing Laplacian differentiator based on quasi-BIC. Reproduced from Ref. [88]. (i) Performing Laplacian differentiator based on TD resonances. Reproduced from Ref. [53].

Surface plasmon polaritons (SPPs) are electronic oscillations at the metal–dielectric interface excited by electromagnetic waves, capable of generating plasmonic resonance with the incident light field [78], [79]. This can manipulate light transmission, making SPPs valuable for nano-photonics, metamaterials, and applications in sensing, imaging, and processing [80], [81], [82], [83]. In 2014, Ruan et al. [84] proposed a method to perform first-order spatial differentiation by exciting SPPs at a metal–dielectric interface. This approach leverages the ability to control the spatial distribution of the electromagnetic field through the interaction with SPPs, effectively allowing for the manipulation of light on a subwavelength scale. Building on this concept, Zhu et al. [50] experimentally demonstrated spatial first-order differentiation and image edge detection based on SPPs. As depicted in Figure 5a, the setup involves a prism and a thin silver film to excite SPPs. By using a specific angle of incidence, SPPs are excited at the metal (silver)–dielectric interface. As these SPPs propagate along the interface, they leak and radiate, interfering with the directly reflected background field. This interference, under critical coupling conditions, enables spatial differentiation operations. The design is relatively simple, relying on the precise tuning of parameters to achieve the critical coupling condition, which is essential for the effectiveness of the method. However, a limitation of this approach is that it facilitates differentiation and edge detection in only one direction, which may restrict its applicability in certain scenarios requiring multidirectional analysis. Yet, the work represents a significant step forward in the development of plasmonic devices for optical computing, showcasing the potential of SPPs in performing complex optical operations.

As a traditional optical device, the grating still possesses formidable capabilities. As shown in Figure 5b, Bykov et al. [85] designed a diffraction grating with one-dimensional periodicity. By exciting guided mode resonance at small incident angles, it is possible to achieve first-order spatial differentiation of an obliquely incident beam near the resonant frequency. By adjusting the geometric features of the grating, Cordaro et al. [86] designed a meta-grating capable of achieving Fano resonance in the visible light spectrum, as shown in Figure 5c. This design can impart optical transfer functions in the momentum space corresponding to first- and second-order spatial differentiation. It enables the processing of input signals or images accordingly. However, since the grating is a one-dimensional array, this spatial differentiation, which relies on the grating, can only be implemented in a single direction.

From the perspective of photonic crystals, gratings can be considered as one-dimensional photonic crystals. Consequently, two-dimensional photonic crystals can readily address the limitations of gratings in implementing optical differentiation, such as realizing a Laplacian differentiator that performs two-dimensional second-order differentiation operations. As depicted in Figure 5d, Guo et al. [20] demonstrated a three-layered photonic crystal slab structure, theoretically proving the implementation of a Laplacian differentiator and isotropic two-dimensional second-order image edge detection. Subsequently, Kwon et al. [51] designed a photonic crystal slab with a triangular lattice hole structure, capable of performing 1st- and 2nd-order analog differentiation operations on input images by exciting Fano resonances, as shown in Figure 5e. According to the optical reciprocity theorem, Zhou et al. [52] designed a photonic crystal composed of cylindrical nano-columns on a glass substrate, as shown in Figure 5f, with these nano-columns embedded in PMMA material that has a refractive index nearly identical to that of the substrate. This photonic crystal structure can excite quasi-BICs under obliquely incident light, meeting the conditions for direct implementation of a Laplacian differentiator in the near-infrared spectrum. Experimentally, it can be integrated with traditional imaging systems, such as commercial optical microscopes and cameras, to achieve two-dimensional second-order edge detection of incident images. Komar et al. [87] utilized a periodic disk metasurface with C_6_ rotational symmetry to simultaneously excite electric and magnetic dipole resonances in the near-infrared region, enabling Laplacian differentiation operations and image edge detection, as illustrated in Figure 5g. Additionally, Pan et al. [88] designed a Laplacian differentiating metasurface with wavelength-scale spatial resolution by exciting quasi-bound states in the continuum, as depicted in Figure 5h. Harnessing the principles of optical resonance and the Green’s function approach, our group Zhou et al. [53] developed a square hollow metasurface Laplacian differentiator, as illustrated in Figure 5i. In this work, the optical transfer function (OTF) required by the optical Laplace operation can be obtained by exciting an angularly selective toroidal dipole (TD) resonance supported by the metasurface. This hollow silicon brick differentiator not only realizes 2D second-order edge detection but also has a numerical aperture close to 0.4 and a broad working wavelength range >100 nm and can be directly integrated with imaging systems. Such meta-device may be potentially applied in the fields of optical sensing, microscopy, machine vision, biomedical imaging, etc.

**Figure 6:**
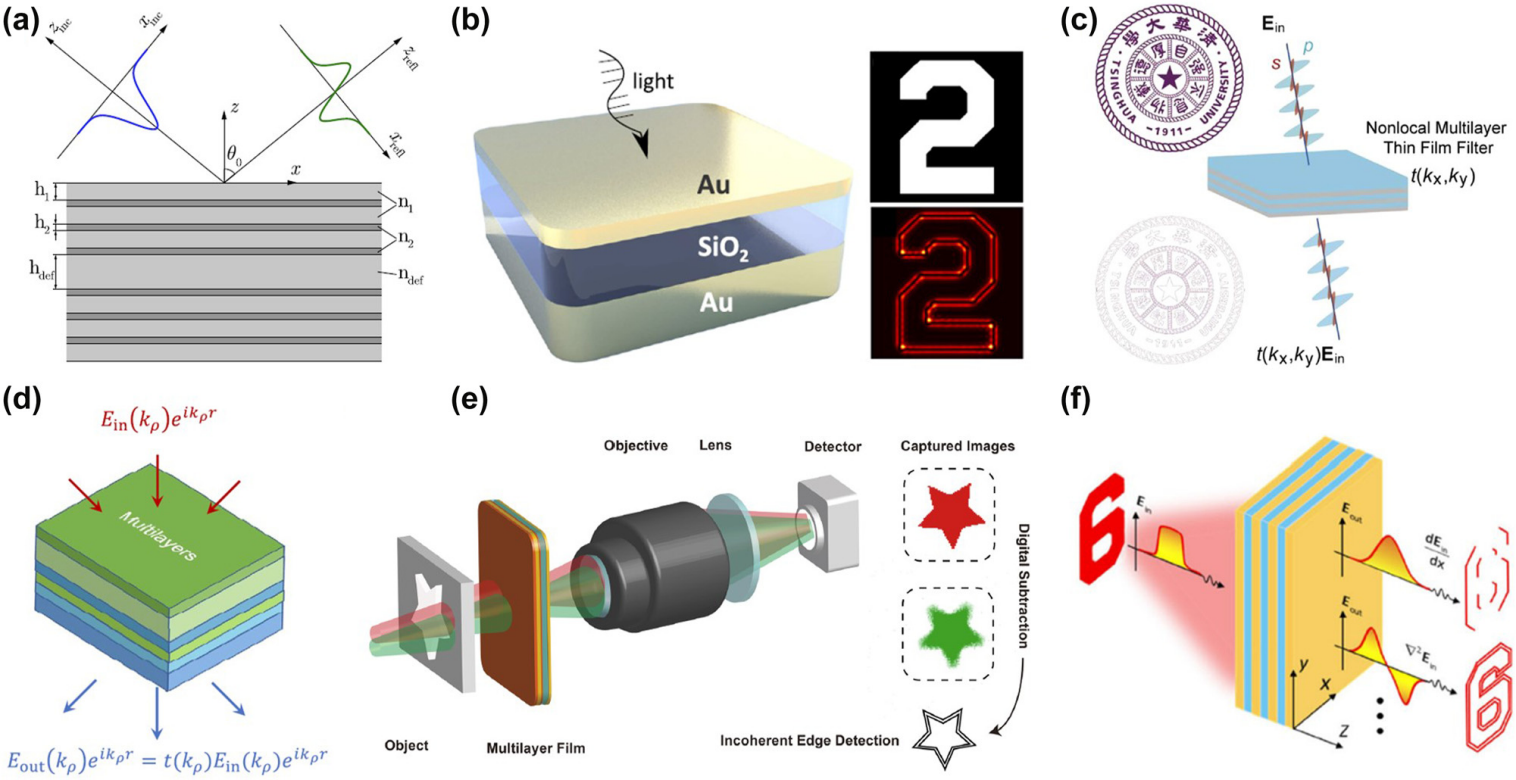
Green’s function approach for optical spatial amplitude differentiator based on the multilayer. (a) Performing spatial differentiation by a phase-shifted Bragg grating in reflection. Reproduced from Ref. [89]. (b) Using metal–insulator–metal (MIM) absorber for Laplace differentiator in reflection. Reproduced from Ref. [90]. (c) Using a transmissive multilayer thin film to perform 2D image differentiation with arbitrary polarization. Reproduced from Ref. [54]. (d) High-NA Laplace differentiation via optimized multilayer films. Reproduced from Ref. [91]. (e) Incoherent optoelectronic two-dimensional differentiation system with the multilayer film. Reproduced from Ref. [92]. (f) Photonic chip acting as a multiple-order spatial differentiator. Reproduced from Ref. [93].

Although photonic crystal structures can pave new avenues for implementing differential operations and image edge detection, optical analog differential computing devices based on the Green’s function can also be realized using multilayer thin-film structures. By leveraging the well-established optimization methods for multilayer films, the capabilities of devices performing differential operations or image edge detection can be further enhanced. For instance, this can be achieved by expanding the operational bandwidth and realizing higher numerical apertures. Doskolovich et al. [89] designed a phase-shifted Bragg grating (PSBG), as depicted in Figure 6a. This PSBG allows to calculate the first-order spatial differentiation at oblique incidence and the second-order differentiation at normal incidence. As an example, it can be able to convert an input 2D Gaussian beam into a 2D Hermite–Gaussian mode. Wesemann et al. [90] demonstrated that, by using the Au/SiO_2_/Au metal–insulator–metal (MIM) absorber structure as depicted in Figure 6b, it is able to achieve a Laplace differentiator in reflection with dual polarization channels. These two examples both utilize guided mode resonance within Bragg gratings to operate in reflection mode, which is not conducive to device integration for the transmission cases.

Due to the mature optimization methods available for multilayer thin-film structures, the execution of differential operations can be achieved without relying on resonance characteristics. As shown in Figure 6c, Jin et al. [54] utilized a particle swarm optimization algorithm to design a transmission-type all-dielectric multilayer thin-film optical filter that can perform Laplacian differentiation on incident light fields of any polarization state, with a numerical aperture of up to 0.3. Xue et al. [91] designed a multilayer thin-film structure with alternating layers of Si and SiO_2_, as depicted in Figure 6d. Through simulations, they verified that the numerical aperture can reach up to 0.98 when implementing Laplacian differentiation operations in the visible light spectrum. Zhang et al. [92] also combined the multilayer thin-film structure directly with traditional electro-imaging systems, extending the execution of optical two-dimensional differentiation operations to incoherent light, as shown in Figure 6e. This device optimizes the optical transfer function at two wavelengths through neural network optimization, achieving polarization-insensitive two-dimensional optical differentiation. Liu et al. [93] proposed a planar chip made of multilayer materials, as illustrated in Figure 6f. Without altering the structural parameters, it can achieve first-order, second-order, and high-order third and fourth differentiation under different polarization states. Leveraging the chip’s differentiation capabilities, when integrated into a traditional imaging system, it enhances the characteristic edges of optical amplitude and phase images, demonstrating the potential to extend its application to standard microscopes. For executing high-order differentiation operations, Huo et al. [94] have also have recently proposed and experimentally validated a Bessel vortex modulated metalens composed of a single complex amplitude metasurface. By predefining the order of the corresponding Bessel vortex, this metalens is capable of performing differentiation operations from the first to the fourth order within a wide frequency band. Furthermore, moreover, this metasurface can create multiple information channels through angular multiplexing, enabling the synchronous execution of multi-order spatial differentiation operations, thus leveraging the parallel processing advantages of optical analog computing.

## 4 Phase imaging and edge enhancement

Most biological samples, such as cells and tissue sections, exhibit weak scattering properties and are nearly transparent under visible light illumination, commonly referred to as phase objects [95], [96]. Traditional optical microscopes primarily rely on changes in optical absorption of the sample for imaging, which results in low contrast for transparent samples, making them difficult to observe accurately. If cells are stained with dyes, it is typically toxic to living cells and can affect their viability [97]. In 1942, Zernike invented the Zernike phase contrast (ZPC) microscope, which uses a specially designed phase plate to convert the phase information of the object into intensity contrast information, thereby greatly improving the imaging contrast of optical microscopes for transparent objects [98], [99]. Zernike was also awarded the Nobel Prize in Physics in 1953 for this technique. Building on this concept, various optical phase imaging methods have been proposed, including common methods such as the spiral phase contrast (SPC), differential interference contrast (DIC), and transport of intensity equation (TIE) [100], [101], [102], [103], [104], [105], [106]. Here, we review the current state of edge enhancement for phase objects using metasurface, employing both the spiral phase contrast method and the Green’s function method.

**Figure 7:**
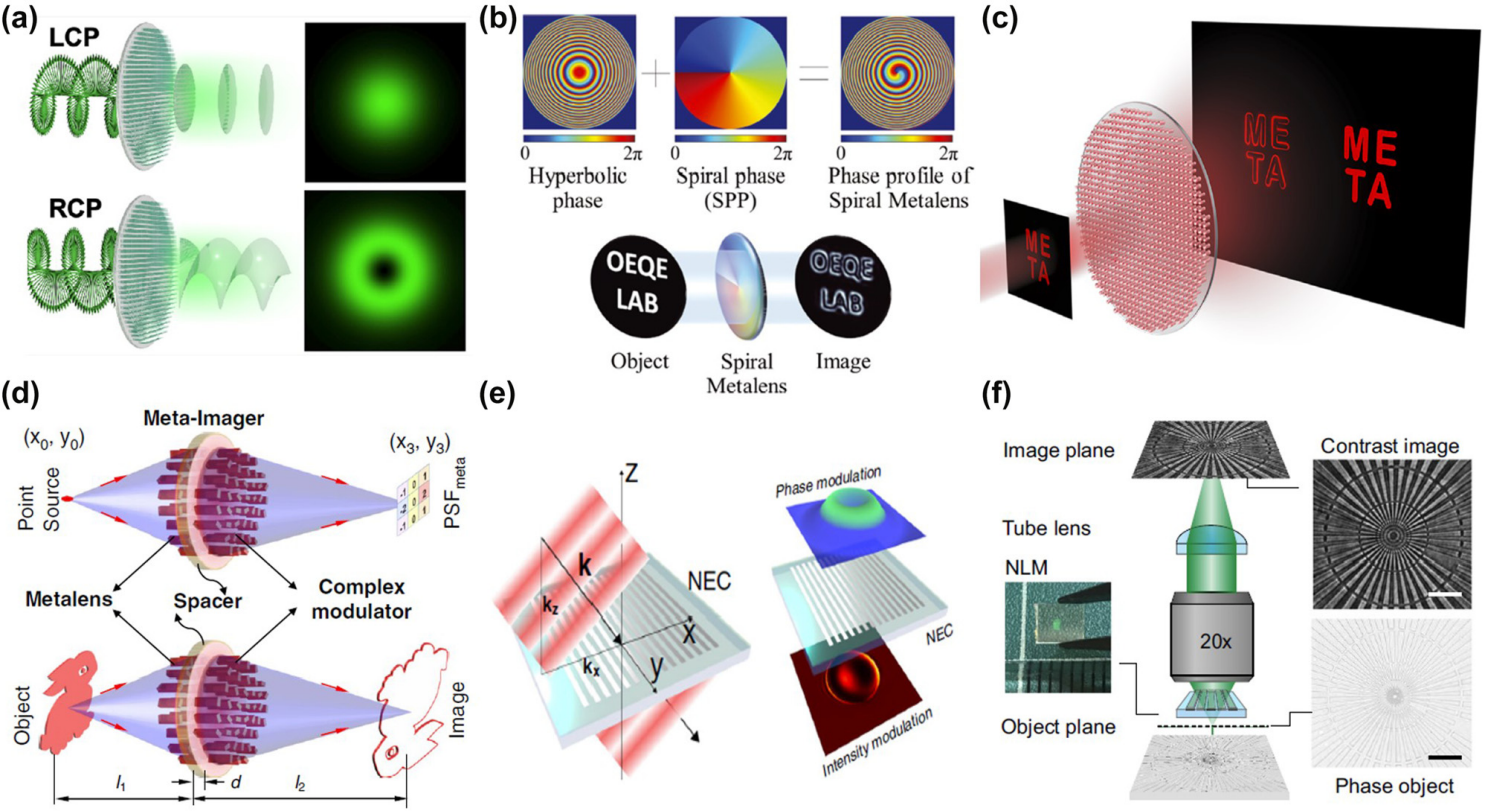
Metasurface-based phase imaging and edge enhancement. (a) Schematic of the photonic spin-multiplexing metasurface for switchable spiral phase contrast imaging. Reproduced from Ref. [55]. (b) Schematic illustration of the spiral metalens. Reproduced from Ref. [107]. (c) Dielectric metasurface for synchronously spiral phase contrast and bright-field imaging. Reproduced from Ref. [108]. (d) Ultracompact meta-imagers for phase imaging and edge enhancement. Reproduced from Ref. [109]. (e) Nanophotonics enhanced coverslip (NEC) for phase imaging and edge enhancement. Reproduced from Ref. [110]. (f) Phase contrast imaging of nonlocal metasurface. Reproduced from Ref. [56].

The spiral phase contrast (SPC) method is similar to the spatial Fourier filtering method, modulating the incident field by acting on the Fourier plane with a spiral phase mask. In image processing, the spiral phase characteristic of vortex light can be used as a filtering mask for edge enhancement, and this method is applied to image processing techniques. Huo et al. [55] developed an optical imaging system based on spin-multiplexing metasurface, which can switch between bright-field imaging and spiral phase contrast imaging modes by changing the spin direction of the incident light, as shown in Figure 7a. The working principle of this metasurface is based on the 4*f* filtering method, loading two phase distributions on different polarization states of the incident light, that is, a constant phase on the left circular polarization (LCP) channel and a spiral phase distribution on the right circular polarization (RCP) channel, thereby forming a polarization-dependent metasurface spatial filter. By adjusting the polarization state of the incident light, dynamic switching between bright-field imaging and isotropic edge enhancement imaging is achieved. Kim et al. [107] combined the phase distribution of a traditional lens with a spiral phase distribution with a topological charge of 1, successfully integrating imaging and edge detection functions into a single-layer metasurface imaging system, as shown in Figure 7b. This method, in practical experimental processes, does not require a pair of lenses in the 4*f* system, thereby greatly enhancing the integrability of the imaging system. Subsequently, Zhang et al. [108] successfully achieving the effect of simultaneous bright-field imaging and isotropic edge enhancement imaging in one imaging process, as shown in Figure 7c.

Furthermore, Fu et al. [109] introduced a compact meta-imager, as depicted in Figure 7d, which is capable of performing arbitrary all-optical convolution calculations. This meta-imager is composed of two parts, a metalens for imaging and a meta-modulator with a complex amplitude for reshaping its point spread function (PSF), thereby achieving convolution kernels of any function. This design not only facilitates one-dimensional and two-dimensional spatial differentiation but also enables real-time, parallel processing of optical and biological sample images. For instance, by placing unstained human cancer cells on the device, Fu and colleagues were able to clearly observe the internal cellular structures. Wesemann et al. [110] have designed a nanostructured coverslip exploits the contrast-forming properties of spatial-frequency filters to image of phase objects, as shown in Figure 7e, that functions similarly to a traditional phase contrast microscope. It serves as an innovative nanophotonics enhanced coverslip (NEC) that can produce high-contrast images of pure phase objects during transmission, which is significant for observing transparent samples such as living cells. Moreover, the principle of phase imaging and the process of implementing a differentiator using the Green’s function method are quite similar.

The authors in the study by Ji et al. [56] proposed a novel phase contrast imaging method by utilizing the nonlocal response of a waveguide resonator metasurface to achieve quantitative phase contrast imaging with precision, as illustrated in Figure 7f. When incident light illuminates such a metasurface structure, most of the light passes directly through the silicon nitride (Si_3_N_4_) waveguide layer. However, at specific wavelengths and angles, the grating can couple the incident light to the quasi-waveguide mode, significantly enhancing the electric field in the silicon nitride layer. The principle based on this phase contrast imaging is possible to direct imaging and edge enhancement of phase objects in the spatial domain. Additionally, no extra complex devices are required. Compared to the conventional implementation of Zernike’s phase contrast imaging, using metasurface enables quantitative phase-contrast imaging without accessing to the back focal plane, thus allowing for the development of more compact imaging systems. In the future, if polarization-independent metasurface is designed to perform broadband phase imaging under white light conditions, which will undoubtedly advance the development of existing microscopic instruments.

## 5 Temporal differentiator and pulse shaping

The aforementioned content primarily introduces the differentiation operation of optical spatial signals, which is mainly based on the 4*f* system or the Green’s function method for filtering light signals. When it comes to temporal differentiation, the focus is on processing the spectral information carried by the optical time pulse envelope, where metasurface and meta-devices still show great potential [111], [112]. Moving forward, we provide a review of the current developments in the field of light field temporal differentiation based on various metasurface.

**Figure 8:**
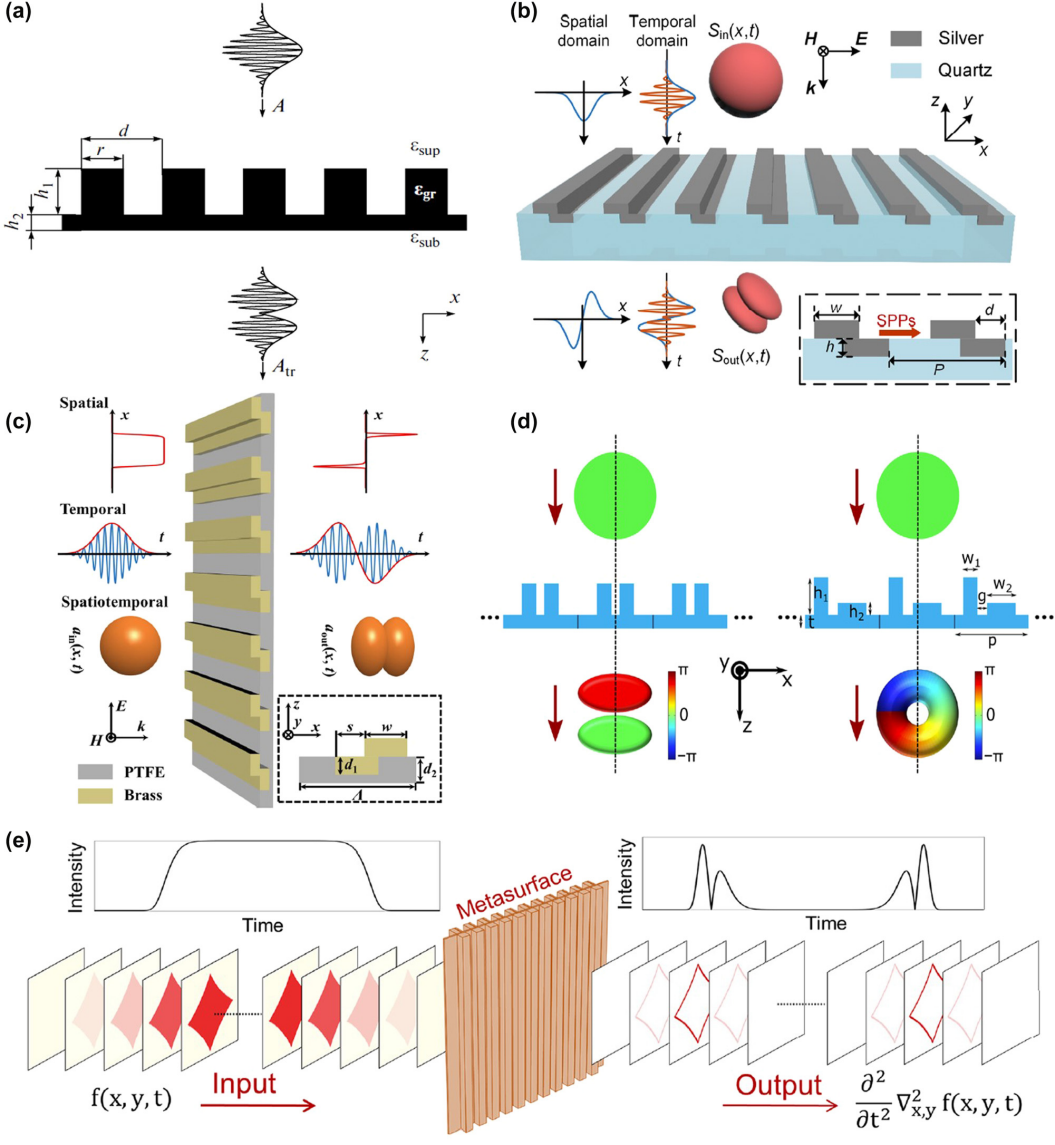
Temporal differentiator based on meta-devices. (a) Resonant diffraction gratings for differentiate a pulsed optical signal. Reproduced from Ref. [113]. (b) Spatiotemporal differentiator based on a bilayer silver grating. Reproduced from Ref. [114]. (c) The analog spatiotemporal differentiator utilizing a bilayer metal grating. Reproduced from Ref. [57]. (d) Schematic illustration of generate spatiotemporal optical vortices (STOVs) with a spatiotemporal differentiator. Reproduced from Ref. [115]. (e) Temporal differentiator based on tailor space-time nonlocality for event-based image processing metasurface. Reproduced from Ref. [116].

Gratings, as a powerful class of optical devices, are capable of not only amplitude modulation but also playing a significant role in temporal differentiation. Bykov et al. [113] investigated the method of temporal differentiation of optical pulse signals using resonant diffraction gratings, as show in Figure 8a. These gratings exhibit sharp variations in reflection and transmission coefficients at specific frequencies. Characterized by minimal longitudinal dimensions and larger transverse sizes, the diffraction process of optical pulses through resonant diffraction gratings was thoroughly analyzed. The authors derived that a specific number of resonance peaks in the grating’s transmission spectrum is essential for achieving high-order temporal differentiation of the pulse envelope. Numerical simulations confirmed the designed gratings’ effectiveness for first-, second-, and third-order temporal differentiation.

Xu et al. [114] designed an optical spatiotemporal differentiator that by breaking the mirror symmetry of a subwavelength double-layer silver grating, as show in Figure 8b. It can simultaneously achieve first-order spatial and temporal differentiation under transmission conditions. Numerical assessment of the output field profile for beams and pulse envelopes revealed spatiotemporal resolutions of 50 fs and 2 μm, respectively. Similarly, Zhou et al. [57] designed an asymmetric metasurface with a phase singularity in the spatiotemporal domain, capable of functioning as a spatiotemporal differentiator in the microwave range, as show in Figure 8c. It is demonstrated that by tailoring the unidirectional excitation of spoof surface plasmon polaritons (SSPPs), this structure can generate a spatiotemporal transfer function necessary for an ideal first-order differentiator in both spatial and temporal domains. Experimentally, spatial edge detection was conducted using a metallic slit, while the temporal differentiation capability was assessed with Gaussian-like temporal pulses of varying widths. Huang et al. [115] designed a spatiotemporal differentiator by breaking the spatial mirror symmetry of one-dimensional periodic nanostructures, as shown in Figure 8d. Such a spatiotemporal differentiator can be regarded as a pulse shaper, capable of generating phase singularities in the spatiotemporal domain. For a normal incident pulse, the differentiator generates a transmitted spatiotemporal optical vortices (STOVs) pulse with transverse orbital angular momentum. Furthermore, the interference of the generated STOVs can be used to detect the sharp changes of pulse envelopes, in both spatial and temporal dimensions.

Nonlocal metasurface, as an ultra-compact, low-power, and high-speed analog image processing platform, not only performs spatial differential operations but also can execute temporal differentiation. Cotrufo et al. [58] designed a resonant waveguide grating (RWG) metasurface made of titanium dioxide (TiO_2_) placed on a quartz substrate. This metasurface, which possesses temporal nonlocality, can perform first-order differentiation on time-domain signals. This means it can be used to identify the instant of times at which a signal is strongly varying. Additionally, this approach is easily scalability and cascaded computation. By cascading such two metasurface, the resulting device performs second-order differentiation. Recently, Esfahani et al. [116] extended the concept of nonlocality engineering to the spatiotemporal domain, by designing a cascaded metasurface as shown in Figure 8e. By adjusting the metasurface’s nonlocal electromagnetic response in space and temporal, they achieved the desired spatiotemporal response to perform analog mixed spatiotemporal differentiation on input images. This functionality can be utilized for event-based edge detection, where the edges of the input image are enhanced only if the image intensity varies over time. Such event-based analog processing necessitates mixed spatiotemporal differentiation, which cannot be accomplished by other forms of spatiotemporal devices. Particularly, in this latter case, the intensity of the filtered image depends on the object speed, which opens interesting applications in the fields of sensing and vibration monitoring.

Metasurface-based temporal and spatiotemporal differentiators hold significant research implications in the fields of modern optics and optoelectronics. These differentiators utilize the unique optical properties of metasurface to realize temporal and spatial differential operations on optical signals, which provide new technical means for the application of ultrafast optical information processing, optical computing, optical identification and coding, and pulse shaping.

## 6 Reconfigurable differentiator

Based on the spatial Fourier transform method approach and the Green’s function (GF) approach, the extraction of morphological information from optical images has become a key technology due to its fast processing. However, most metasurface-based differentiators are static filters, with their transfer functions being statically fixed after fabrication, thus limiting their multifunctionality, dynamic capabilities, and practical applications. The dynamic modulation of metasurface can be achieved through mechanical stretching, electrical tuning, thermal regulation, optical modulation, chemical modulation, and the use of active materials [117], [118], [119], [120], [121], [122]. Particularly, liquid crystal materials and phase-change materials, such as Ge_2_Sb_2_Te_5_ (GST) or vanadium dioxide (VO_2_), play a significant role in the realization of dynamically tunable metasurface [123], [124], [125], [126], [127], [128], [129]. These methods can also be applied to achieve dynamic optical differentiation and corresponding dynamic image edge detection processing.

**Figure 9:**
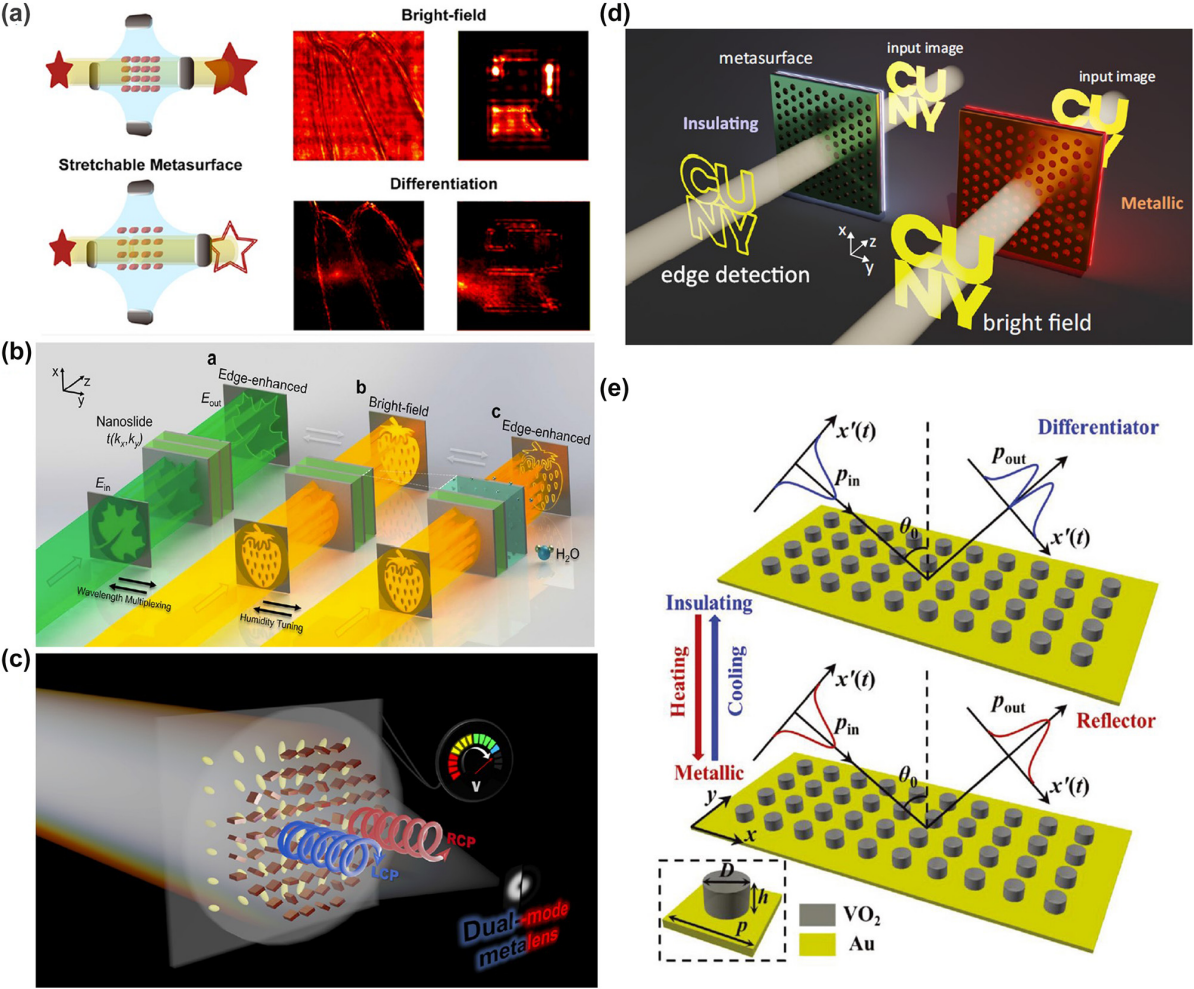
Reconfigurable differentiator based on metasurface. (a) Tunable metasurface for bright-field imaging and differentiation under different strain. Reproduced from Ref. [130]. (b) Hydrogel-scalable nanoslide for switchable optical spatial-frequency processing. Reproduced from Ref. [131]. (c) Schematic of the proposed electrically tunable dual-mode metalens. Reproduced from Ref. [132]. (d) Reconfigurable image processing metasurface with phase-change materials. Reproduced from Ref. [59]. (e) The reconfigurable temporal optical signal processor based on the phase-change metasurface. Reproduced from Ref. [133].

As illustrated in Figure 9a, Zhang et al. [130] presented a reconfigurable metasurface that can dynamically adjust the period of the metasurface units embedded in PMMA through mechanical stretching. This allows for the execution of various image processing tasks on the input image, including bright-field imaging, low-pass and high-pass filtering, and second-order differentiation for edge detection. Such a dynamically tunable metasurface can be directly integrated with standard coherent imaging systems and operates with a numerical aperture of up to 0.25 and a bandwidth exceeding 60 nm. Dai et al. [131] proposed a method for switchable edge-enhanced imaging and bright-field imaging by utilizing a tunable hydrogel-scalable nanoslides, as depicted in Figure 9b. By employing a multilayer structure of metal and hydrogel, the nanoslides directly manipulate optical spatial frequencies in the wave vector domain, and due to cavity-induced wavelength sensitivity, they exhibit opposite image processing in different wavelength channels. More intriguingly, by controlling the ambient humidity, the angular-dependent optical response of these nanoslides can be effectively adjusted for dynamic edge-enhanced imaging, owing to the water absorption and swelling of the hydrogel. Moreover, the nanoslides are easily fabricated at a large scale and integrated into compact imaging systems, such as biological microscopes. Additionally, Badloe et al. [132] design and experimentally realized a dual-mode metalens integrated with liquid crystal units, as shown in Figure 9c, which can be electrically switched between bright-field imaging and edge-enhanced imaging on a millisecond timescale. This dual-mode metalens combines geometric phase and propagation phase, physically encoding the required phase profiles to operate at visible wavelengths. The two distinct meta-lens phase profiles distributions include a conventional hyperbolic metalens for bright-field imaging and a spiral metalens with a topological charge of +1 for edge-enhanced imaging. Such dynamically tunable devices can be utilized *in vivo* observation and monitoring of the cell response and drug screening.

Recently, as shown in Figure 9d, Cotrufo et al. [59] demonstrated a type of passive edge-detection metasurface operating in the near-infrared regime whose response can be drastically modified by temperature variations smaller than 10 °C around a CMOS-compatible temperature of 65 °C. The structure of this metasurface primarily consists of a thin layer of vanadium dioxide placed beneath a specially patterned photonic crystal slab. The dynamic reconfigurability is enabled by the phase transition of vanadium dioxide from an insulating to a metallic state, which significantly modifies the nonlocal response of the metasurface. Importantly, this reconfigurability is accompanied by excellent performance metrics, such as numerical aperture, efficiency, isotropy, and polarization independence, and is combined with simple geometric structures compatible with large-scale manufacturing. It holds potential applications in augmented reality, remote sensing, and biomedical imaging. Additionally, Zhou et al. [133] proposed a reconfigurable spatiotemporal optical signal processor employing the phase-change material vanadium dioxide (VO_2_), as illustrated in Figure 8d. This approach further broadens the processing bandwidth and enriches operational functionalities. The spatiotemporal differentiator can switch between a first-order differentiator and an efficient reflector by controlling the operating temperature, thereby enabling precise design of the spatiotemporal transfer function to meet the requirements of ideal first-order differentiation and bright-field imaging. This method facilitates high-precision spatial and temporal signal processing, such as inspecting Gaussian signal variations, and holds significant potential for applications in remote sensing and rapid imaging processing.

## 7 Nonlinear differentiator

Current optical analog differentiator based on metasurface primarily relies on materials with time-invariant optical responses and limited to linear operations. Based on the nonlinear properties of metasurface, designing devices capable of nonlinear differentiation and related edge detection is of great significance in practical applications such as night vision imaging [134], [135], [136], [137].

**Figure 10:**
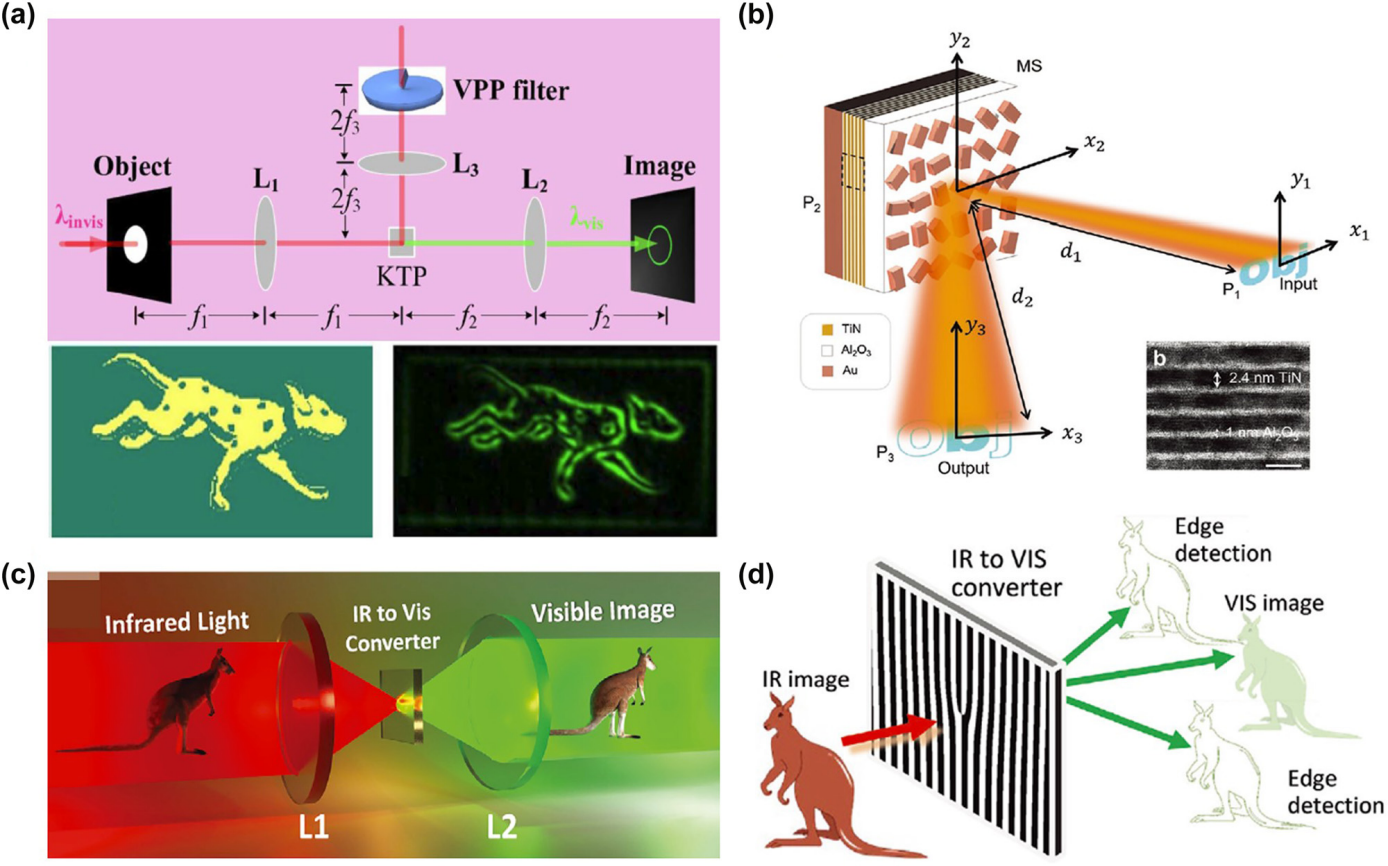
Nonlinear differentiator based on metasurface. (a) Schematic diagram of nonlinear spiral phase contrast imaging. Reproduced from Ref. [143]. (b) Nonlinear computational edge detection metalens. Reproduced from Ref. [144]. (c) and (d) Infrared (IR) to visible (VIS) up-conversion for image processing. Reproduced from Ref. [60].

In the field of nonlinear imaging based on meta-devices, to address the drawbacks of traditional infrared image detectors, such as low efficiency, poor resolution, and complex equipment, frequency conversion methods are commonly employed to shift infrared image information to the visible light spectrum [138], [139], [140], [141], [142]. Subsequently, high-efficiency, high-resolution, and simple visible light detectors are used for image acquisition. Despite significant experimental and theoretical progress in this area, nonlinear differentiator imaging with invisible light is still lacking. As shown in Figure 10a, Qiu et al. [143] construct a nonlinear spatial filter by equivalently imprinting the vortex phase plate onto the potassium titanyl phosphate crystal using second harmonic generation (SHG). The phase or intensity objects are illuminated with 1,064 nm infrared light. Then, the combination of such nonlinear filter with SHG in the Fourier domain, yet highly efficient SPC imaging, leading to a visible edge enhancement with invisible illumination. This scheme could find direct applications in infrared monitoring.

Zhou et al. [144] proposed a single nonlinear metalens with an illumination intensity-dependent coherent transfer function (CTF) as depicted in Figure 10b. The nonlinear metalens consisting of nanoantenna structures with a static geometric phase and a nonlinear metallic quantum well layer (a stack of TiN/Al_2_O_3_) offering an intensity-dependent dynamic phase result in a continuously tunable CTF. In this context, the nonlinear effects primarily originate from the TiN quantum wells possess giant Kerr nonlinearity. Experiments demonstrated that the metalens was capable of performing various computational imaging functions in reflection mode without the need for any additional imaging elements, including bright-field imaging and edge detection. This nonlinear metalens may lead to important applications in optical neural networks and parallel analog computing. Molina et al. [60] have designed a nonlocal lithium niobate resonant metasurface with a high-quality factor. By integrating spatial Fourier transfer approach and a nonlinear up-conversion process, they demonstrated the conversion of infrared imaging to visible light imaging, as shown in Figure 10c. Despite the strong nonlocality of the metasurface, high conversion efficiency and high-resolution imaging can still be achieved. In a single shot, the up-conversion process of the metasurface allows the metasurface to perform direct infrared imaging and edge detection image processing in different diffraction channels simultaneously, as shown in Figure 10d. This nonlinear metasurface is expected to find important applications in future compact night vision devices, sensor equipment, and room-temperature multicolor imaging.

## 8 Conclusions and outlook

Nowadays, meta-devices, represented by metasurface, have gained significant value in nanophotonics, playing a crucial role in explaining scientific discoveries, technological advancements, and novel physical phenomena. Their inherent thin and light characteristics, coupled with their powerful light field manipulation capabilities, can greatly enhance the versatility and integration of optical systems. In this review, we summarize the latest developments and emerging application trends of meta-devices composed of gratings, multilayer films, and metasurface as spatial optical analog differentiators. Starting from two fundamental design principles based on the spatial Fourier transform approach and the Green’s function approach, we systematically review the physical mechanisms of these meta-device-based spatial optical differentiators in amplitude differentiation, phase differentiation, and temporal differentiation. We review their applications in image edge detection, edge enhancement, and beam shaping. Additionally, we also analyze the progress of reconfigurable differentiators and differentiators implemented using nonlinear principles.

**Figure 11:**
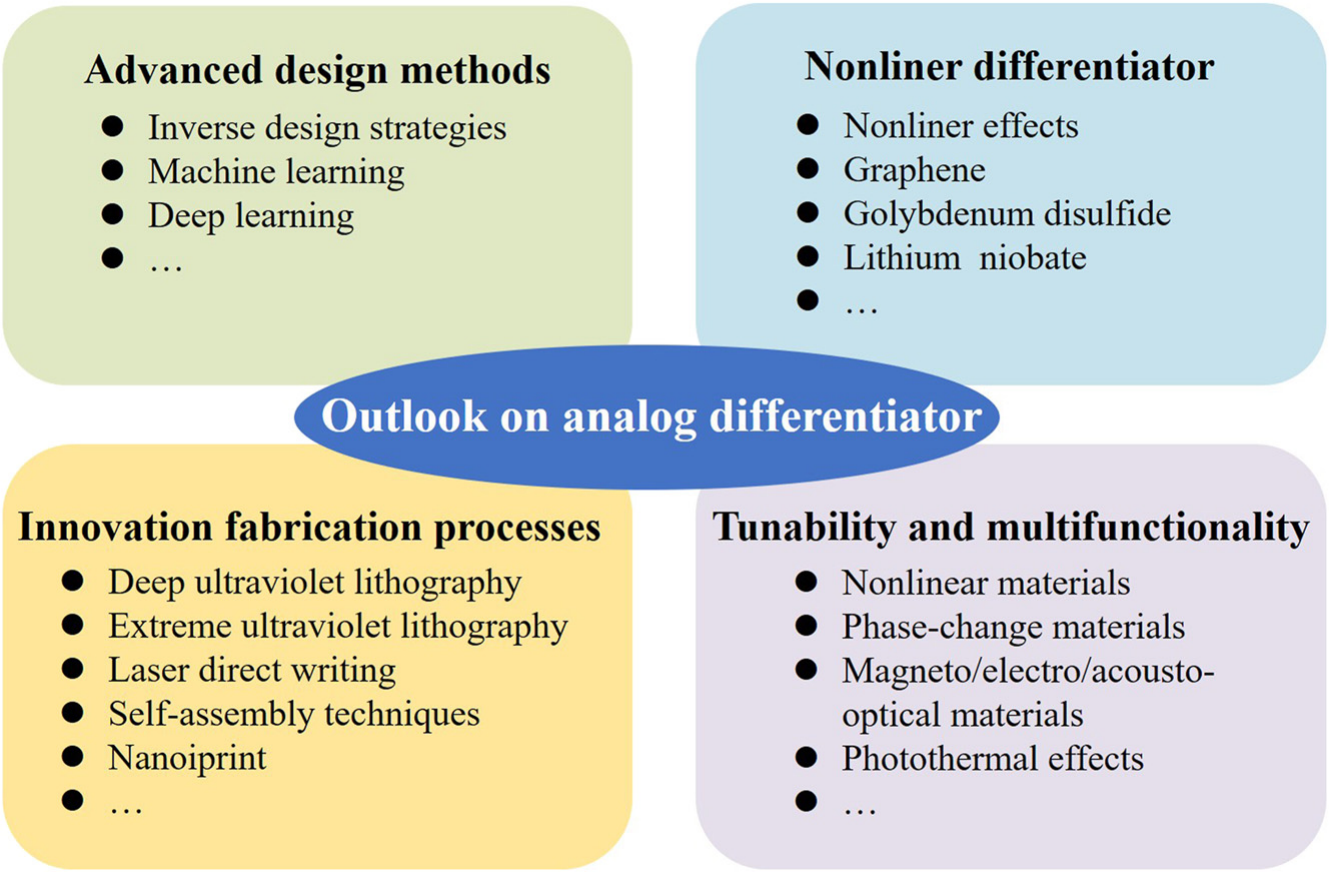
The outlook on analog differentiator empowered by meta-devices from advanced design methods, nonlinear differentiator, innovation fabrication processes, and tunability and multifunctionality.

In general, spatial optical analog differential computation and information processing – encompassing applications such as image edge detection, edge enhancement, and beam shaping – represent distinct fields. Each has its own developmental focal points. As the demand for increased data acquisition and processing speed grows, along with an urgent need for miniaturization and diversification of device functions. The evolution of meta-devices has played a significant role in propelling the development and integration of optical simulation and information processing. All-optical analog differential devices based on meta-devices offer unparalleled advantages in terms of energy consumption, computation speed, parallel processing capabilities, and storage when it comes to implementing differential computation and information processing. This renders them valuable for applications in advanced microscopic imaging, autonomous driving, machine vision, and laser processing. Despite the significant advancements in meta-device-based optical analog differential computation and information processing, such as edge detection, challenges in structural design, manufacturing processes, material performance, and dynamic control. In those regard, looking ahead, several potential solutions and research directions for future studies can be anticipated, as shown in Figure 11.

Firstly, to achieve integration with other all-optical analog computing functions or higher-order differential operations, meta-devices need to have smaller unit structures, more complex structural combinations, and larger actual apertures. This poses new requirements for the development of precise design methods and optimization model algorithms. In this context, the combination of advanced artificial intelligence technologies such as inverse design methods, machine learning, and deep learning provides new ideas and platforms for solving this problem [145], [146], [147], [148], [149], [150], [151]. Various optimization techniques can be employed to find the theoretically optimal structural design.

Secondly, the fabrication area of meta-device-based analog differentiators is currently limited to the micrometer or millimeter scale. Applications at the centimeter scale are still in the laboratory stage, and the costs are high, making it uneconomical and inefficient for industrial production. At present, the manufacturing of meta-devices primarily relies on electron-beam lithography (EBL). There is a need to accelerate the development and innovation of advanced micro- and nano-fabrication technologies, such as deep ultraviolet (DUV) lithography, extreme ultraviolet (EUV) lithography, laser direct writing, self-assembly techniques, and nanoimprint lithography, to fabricate high-precision metasurface structures that can meet the demands of large-scale industrialization [152], [153], [154]. Furthermore, due to the limitations of current fabrication technologies, analog differentiators based on meta-devices are mainly applied in photography and microscopy. If meta-devices capable of replacing traditional astronomical telescopes and complex imaging processing systems, including those with image differentiation processing, were realized, it would make a significant contribution to the field of astronomical imaging [155], [156], [157].

Then, the majority of analog differentiators currently rely on the framework of linear system theory. The research and application of meta-devices for nonlinear analog computation, by incorporating the physical mechanisms of nonlinear effects, are still in their infancy [158]. Here, two potential approaches are being explored. One is to utilize materials inherently possessing nonlinear characteristics for the fabrication of analog differentiators, such as materials with gain and excitons like graphene and molybdenum disulfide [159], [160]. The other is to design meta-devices that can excite optical nonlinear effects and are applicable for the creation of analog differentiators [161], [162], [163]. Additionally, the application of these nonlinear analog differentiators or nonlinear analog computational devices in the fields of quantum computing and quantum imaging could be further investigated [74], [164], [165], [166], [167].

Finally, in most practical photonic applications, tunability, multifunctionality, and integrability are still highly sought after. Actively tunable multifunctional spatial analog differentiators and multifaceted information processing methods are also particularly important. For instance, the integration of nonlinear materials or phase-change materials with meta-devices could promote the development of multifunctional devices [59]. It is also possible to utilize magneto-optical materials, electro-optical materials, acousto-optical materials, photochemical materials, or optomechanical methods for the excitation under external conditions [168]. These meta-device-based flexible and controllable analog differential computations technologies can serve as independent computational units to be integrated into the large scale optical intelligent computing systems, which are used for the direct extraction of features from target objects, and are expected to further enhance the overall computational capacity of the system [169], [170]. Furthermore, meta-devices with photothermal effects can be designed to dynamically extend current visible or near-infrared analog differentiators and imaging systems into the information processing domain of long-wave infrared (LWIR) regime and beyond [171], [172].
